# TRPV1 enhances the afferent response to P2X receptor activation in the mouse urinary bladder

**DOI:** 10.1038/s41598-017-18136-w

**Published:** 2018-01-09

**Authors:** Luke Grundy, Donna M. Daly, Christopher Chapple, David Grundy, Russ Chess-Williams

**Affiliations:** 1 Centre for Urology Research, Faculty of Health Science and Medicine, Bond University, Gold Coast, Queensland 4229 Australia; 2 Department of Pharmacy and Biomedical Science, University of Central Lancashire, Preston, PR1 2HE UK; 3 Royal Hallamshire Hospital, Glossop Road, Sheffield, S10 2JF UK; 4 Department of Biomedical Science, University of Sheffield, Sheffield, S10 2TN UK; 5 Visceral Pain Group, University of Adelaide, SAHMRI, Adelaide, Australia

## Abstract

Both TRPV1 and P2X receptors present on bladder sensory nerve fibres have been implicated in mechanosensation during bladder filling. The aim of this study was to determine possible interactions between these receptors in modulating afferent nerve activity. In wildtype (TRPV1^+/+^) and TRPV1 knockout (TRPV1^−/−^) mice, bladder afferent nerve activity, intravesical pressure, and luminal ATP and acetylcholine levels were determined and also intracellular calcium responses of dissociated pelvic DRG neurones and primary mouse urothelial cells (PMUCs). Bladder afferent nerve responses to the purinergic agonist αβMethylene-ATP were depressed in TRPV1^−/−^ mice (p ≤ 0.001) and also in TRPV1^+/+^ mice treated with the TRPV1-antagonist capsazepine (10 µM; p ≤ 0.001). These effects were independent of changes in bladder compliance or contractility. Responses of DRG neuron to αβMethylene-ATP (30 µM) were unchanged in the TRPV1^−/−^ mice, but the proportion of responsive neurones was reduced (p ≤ 0.01). Although the TRPV1 agonist capsaicin (1 µM) did not evoke intracellular responses in PMUCs from TRPV1^+/+^ mice, luminal ATP levels were reduced in the TRPV1^−/−^ mice (p ≤ 0.001) compared to wildtype. TRPV1 modulates P2X mediated afferent responses and provides a mechanistic basis for the decrease in sensory symptoms observed following resiniferatoxin and capsaicin treatment for lower urinary tract symptoms.

## Introduction

As the bladder fills, the degree of distension is detected by mechanosensitive ion channels located either on sensory nerve terminals or on the specialised epithelial lining of the bladder, the urothelium. This evokes an afferent signal that is conveyed to the CNS via the dorsal root ganglia. Initially this signal lies below the level of consciousness, supplying the autonomic reflexes responsible for controlled bladder filling. As bladder volume increases, however, a sensation of fullness and a desire to void is perceived. In continent individuals this desire can be deferred until it is convenient to empty the bladder, however in patients with lower urinary tract conditions such as overactive bladder syndrome (OAB), bladder pain syndrome (BPS) or interstitial cystitis (IC), this sensation is difficult to defer, occurs when the bladder is not yet full and can even be accompanied by pain (BPS/IC) suggesting that a defect in the ability of the bladder to detect filling may underlie these conditions.

Sensory innervation from the bladder is conveyed by myelinated low threshold Aδ fibres and unmyelinated high threshold C fibres carried in the pelvic, hypogastric and pudendal afferent nerves^1^. However, the complex mechanisms which determine how the afferent nerves detect bladder filling remain poorly understood, limiting the development of effective treatments for these bladder sensory defects.

There is compelling evidence that purinergic receptors play an active role in mechanosensation of the bladder in rodents. Firstly bladder projecting afferents express a number of P2X puringeric receptors, of which P2X2 and P2X3 are the main subtype. Secondly, ATP is able to activate pelvic nerve afferents arising from the rat urinary bladder and initiate bladder overactivity^2,3^. Thirdly, the P2X1 and P2X3 agonist, αβMethylene-ATP increases firing in both nociceptive and non-nociceptive populations of bladder afferents^4^. Finally, it has been shown that stretch or distension of the urothelium evokes the graded release of non-neuronal ATP^5^ which activates purinergic receptors located on the afferent terminal to control afferent firing. Previous studies using P2X3 or P2X2/3 null mice found profound attenuation of the afferent response to bladder filling, increased bladder capacity and decreased voiding frequency, supporting a role for ATP in bladder sensation^5,6^. Purinergic receptors are likely to play a role in human urinary bladder physiology and pathophysiology, as P2X receptors are expressed in bladder smooth muscle^7^ and have been shown to mediate a non-adrenergic non-cholinergic component of contraction from patients with idiopathic detrusor instability^8^. P2X receptors have also been identified in myofibroblasts, urothelium, and sub-urothelial nerve fibres of human bladders^9–11^.

The transient receptor potential (TRP)V1 receptor is a member of the TRP superfamily, activated by noxious heat ≥ 43 °C, pH ≤ 6.0, and the vanilloids capsaicin and resiniferatoxin. TRPV1 is mostly confined to unmyelinated, small diameter primary afferent fibres and has been consistently implicated in nociception, and inflammatory pain^12,13^. In the urinary tract, TRPV1 is predominantly expressed on primary sensory afferent nerve fibres, whilst contradictory evidence both support and negate a functional role for TRPV1 in the urothelium^14–20^.

There is evidence to suggest that the TRPV1 receptor contributes to afferent nerve responses during bladder filling. TRPV1 knockout (TRPV1^−/−^) mice have reduced afferent responses to bladder distension, reduced reflex bladder activity and increased bladder capacity^21,22^. Moreover, application of capsaicin or RTX causes activation of bladder sensory nerves and increased reflex activity^21,23^, while systemic administration of a TRPV1 antagonist increases micturition threshold volume and decreases bladder contraction amplitude^24^. Previous studies have also shown that in experimental models of cystitis, genetic deletion or pharmacological blockade of TRPV1 prevents bladder hyperactivity^25–27^. However, TRPV1 is not considered to be a mechanically gated ion channel and thus the mechanism by which it is able to alter bladder sensitivity to filling remains unclear.

Previous clinical studies have shown that P2X3 immunoreactivity is significantly reduced in patients who respond positively to intravesical RTX therapy suggesting TRPV1 and P2X3 receptor expression may be coupled^28^. Moreover, in cultures of human urothelial cells TRPV1 was identified at both mRNA and protein level and stimulation led to ATP release^20^, consistent with earlier studies in rodents^22,29^. Such studies implicate the urothelium in bladder sensory signalling, which we expanded upon here, in order to better understand the role of TRPV1 in modulating purinergic evoked bladder afferent activity in the mouse.

## Results

### Afferent nerve activity

**Figure 1:**
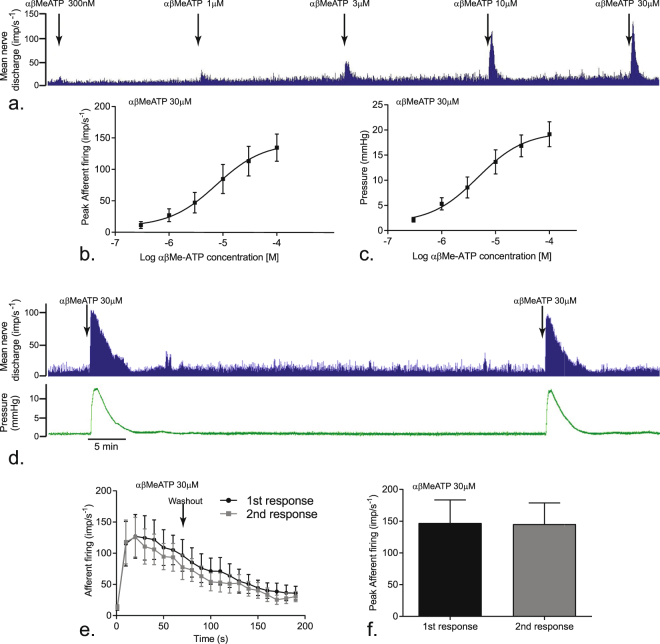
Bladder afferent nerves respond to αβMe-ATP. (**a**) Experimental trace of afferent nerve activity in response to increasing concentrations of αβMe-ATP applied for 60 s before washout; (**b**) concentration-response curve of afferent nerve activity to αβMe-ATP; (**c**) concentration-response curve of intravesical pressure (contraction) to αβMe-ATP; (**d**) afferent nerve and intravesical pressure responses to repeat applications of αβMe-ATP (30 µM); (**e**) afferent nerve time course response to repeated applications of αβMe-ATP (30 µM) applied for 60 s before washout; (**f**) peak afferent firing in response to repeat application of αβMe-ATP (30 µM). Data shown are mean ± SEM (n = 9).

Addition of αβMe-ATP (300 nM–100 µM) produced concentration-related increases in afferent nerve activity and intravesical pressure, indicating detrusor contraction (Fig. 1a,b,c). The afferent response was characterised by an initial rapid component followed by a period of more sustained firing before returning to baseline over 2–3 minutes. Application of αβMe-ATP (30 µM) initiated a pronounced detrusor contraction (Fig. 1d). The maximal firing and time course profiles in response to repeated application of αβMe-ATP were reproducible (Fig. 1e,f) (p ≥ 0.05, n = 9).

**Figure 2:**
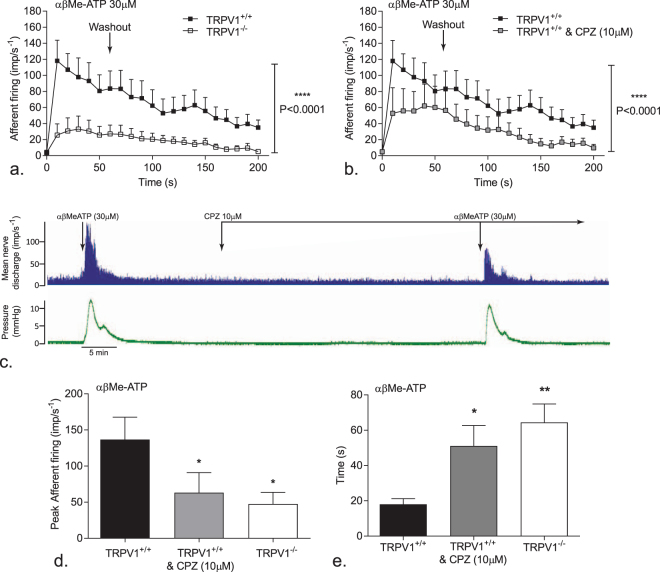
TRPV1 has a role in P2X mediated bladder afferent signaling. (**a**) Afferent nerve response to αβMe-ATP(30 µM), applied for 60 s, in bladder preparations from TRPV1^+/+^ and TRPV1^−/−^ mice. (**b**) Afferent nerve response to repeated applications of αβMe-ATP for 60 s in bladders from TRPV1^+/+^ mice in the presence and absence of capsazepine; Data shown are mean ± SEM, two-way ANOVA with Tukeys post-hoc test ****p ≤ 0.0001 (n = 9). (**c**) Afferent nerve and intravesical pressure responses to repeat applications of αβMe-ATP in TRPV1^+/+^ mice prior to and following incubation with capsazepine. (**d**) Peak afferent nerve responses to αβMe-ATP (30 µM) in TRPV1^+/+^ mice in the presence and absence of capsazepine, and TRPV1^−/−^ mice; (**e**) Time taken to reach maximum afferent nerve impulse frequency in response to αβMe-ATP (30 µM). Data shown are mean ± SEM, one-way ANOVA with Tukeys post-hoc test *p < 0.05, **p ≤ 0.01 (n = 9).

The afferent response to αβMe-ATP was significantly attenuated in TRPV1^−/−^ mice compared to TRPV1^+/+^ control mice (p ≤ 0.0001, n = 9) (Fig. 2a). In order to examine this interaction further, the responses to αβMe-ATP in TRPV1^+/+^ mice were also examined in the absence and presence of the TRPV1 receptor antagonist capsazepine (Fig. 2b,e). Capsazepine (10 µM) had no effect on bladder pressure or baseline afferent firing but significantly attenuated the response to αβMe-ATP (p ≤ 0.0001, n = 9) similar to that seen in TRPV1^−/−^ mice (p < 0.05, n = 9) (Fig. 2b,c,d). Moreover, the time taken to reach maximal afferent firing was delayed in preparations from the TRPV1^−/−^ mouse or following application of capsazepine (18 ± 3.2 s vs 64.4 ± 10.4 s and 51.1 ± 11.6 s respectively p < 0.05 for both changes, n = 9, Fig. 2e).

**Figure 3:**
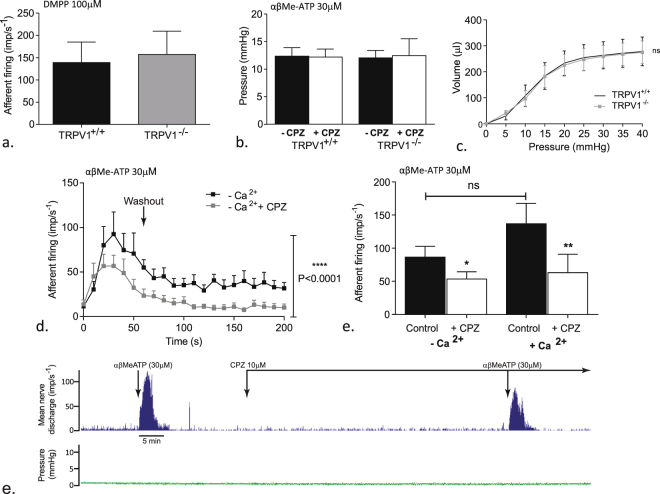
TRPV1 is coupled to P2X mediated afferent firing. (**a**) Peak afferent nerve responses to bath applied DMPP (100 µM) in TRPV1^+/+^ and TRPV1^−/−^ mice. (**b**) Bladder contraction responses to αβMe-ATP (30 µM) in the presence (white bars) and absence (black bars) of capsazepine in TRPV1^+/+^ and TRPV1^−/−^ mice. (**c**) pressure/volume relationship within the bladder when distended with saline in TRPV1^+/+^ and TRPV1^−/−^ mice. (**d**) Afferent nerve response to repeated applications of αβMe-ATP for 60 s in calcium free modified krebs solution with, or without, capsazepine (10 µM); Data shown are mean ± SEM, two-way ANOVA with Bonferroni post-test ****p ≤ 0.0001 (n = 7). (**e**) Afferent response to αβMe-ATP (30 µM) in calcium free modified Krebs solution with or without capsazepine. Data shown are mean ± SEM, one-way ANOVA with Tukeys post-hoc test ns p ≥ 0.05, *p < 0.05 (n = 7).

To determine if the decreased response to αβMe-ATP was due to a general change in neuronal excitability in the TRPV1^−/−^ mice, peak afferent nerve responses to the nicotinic agonist DMPP was examined (Fig. 3a). In contrast to αβMe-ATP, DMPP had a similar effect in TRPV1^+/+^ and TRPV1^−/−^ mice (139.1 ± 46.1imp s^−1^ n = 12 and 157.2 ± 52.3imp s^−1^ respectively, n = 7, p ≥ 0.05).

The afferent nerve response to αβMe-ATP occurred concurrent with a moderate contraction of the detrusor smooth muscle. It is possible therefore that the changes in afferent nerve firing in the TRPV1^−/−^ mice was secondary to altered detrusor muscle activity. However, this would appear not to be the case since there was no significant difference in αβMe-ATP induced bladder contraction between preparations from TRPV1^+/+^ and TRPV1^−/−^ mice despite markedly different afferent firing profiles. Furthermore capsazepine (10 µM) failed to alter contractile responses to αβMe-ATP (p ≥ 0.05, n = 4) (Fig. 3b). Bladder compliance was assessed by the pressure/volume relationship during distention. Compliance was also unaffected by the loss of functional TRPV1 receptors (Fig. 3c). To further examine the potential link between afferent firing and contractile activity the response to αβMe-ATP was compared under calcium free conditions. Removal of calcium completely abolished αβMe-ATP induced contraction (Fig. 3f). However, a robust afferent response to αβMe-ATP was maintained in calcium free conditions (Fig. 3d,e,f). Moreover, application of capsazepine still attenuated the afferent and peak afferent response to αβMe-ATP in calcium-free buffer (Fig. 3d,e,f; 86.6 ± 16.3imp^−s^ Vs 53.6 ± 10.8imp^−s^, (p < 0.05, n = 7).

### Responses in the DRG

**Figure 4:**
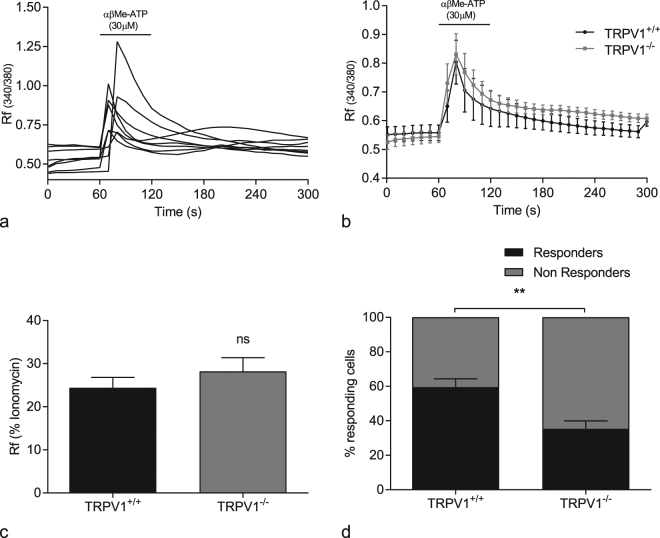
The role of TRPV1 in DRG responses to P2X agonists. (**a**) Individual DRG neurons respond to αβMe-ATP (30 µM), applied for 60 s before washout, with an increase in intracellular calcium; (**b**) mean response of DRGs to αβMe-ATP (30 µM) from TRPV1^+/+^ and TRPV1^−/−^ mice; (**c**) peak intracellular calcium response of DRGs to αβMe-ATP (30 µM) from TRPV1^+/+^ and TRPV1^−/−^ mice; (**d**) percentage of DRG neurons responding (black bar) and non-responding (grey bar) to αβMe-ATP (30 µM); Data shown are mean ± SEM, two-tailed unpaired t-test ^**ns**^ p ≥ 0.05, **p ≤ 0.01 (n = 12, N = 80).

A proportion of DRG neurons responded to αβMe-ATP with an increase in intracellular calcium (Fig. 4a). A subset of DRG neurons from WT mice responded to capsaicin (around 60% Fig. 4b), however, as expected, DRG neurons from TRPV1^−/−^ failed to exhibit responses to capsaicin. There was no significant difference in maximal αβMe-ATP (30 µM) induced calcium rise in DRG neurons (24.4 ± 2.4% Vs 28.1 ± 3.2%) from TRPV1^+/+^ and TRPV1^−/−^ mice respectively (p ≥ 0.05, N = 12, n = 80) (Fig. 4c). However, the percentage of DRG neurons responding to αβMe-ATP was significantly lower in the TRPV1^−/−^ mice compared to TRPV1^+/+^ (fig. 4d) (59.4 ± 4.9 Vs 36.3 ± 4.1%, p ≤ 0.01, N = 12).

### Primary mouse urothelial cells

**Figure 5:**
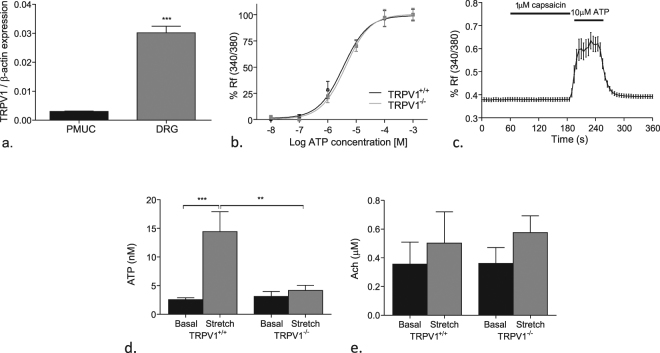
The effect of TRPV1 on urothelial responses to P2X stimulation. (**a**) Relative expression of TRPV1 in PMUC and pelvic DRG neurons;; Data are mean ± SEM, two tailed un-paired t-test ***p ≤ 0.001 (n = 3); (**b**) normalised concentration response curve of PMUC intracellular calcium to ATP in TRPV1^+/+^ and TRPV1^−/−^ mice (n = 6); (**c**) change in intracellular calcium levels of PMUCs to capsaicin and ATP; (**d**) intraluminal ATP and (**e**) acetylcholine release from TRPV1^+/+^ and TRPV1^−/−^ mouse bladders; Data are mean ± SEM, one-way ANOVA with Tukeys post-hoc test **p ≤ 0.01, ***p ≤ 0.001 (n = 6).

In order to examine how the urothelium might contribute to purinergic-TRPV1 signalling, experiments were performed with PMUCs and compared with isolated pelvic DRG neurons. mRNA for the TRPV1 receptor was expressed in both PMUCs and DRGs (Fig. 5a). However, the levels of expression were significantly greater in DRG neurons than PMUCs (p ≤ 0.01, n = 3). PMUCs responded to the purinergic agonist ATP with an increase in intracellular calcium in a concentration-dependent manner (Fig. 5b) with pEC50 values of 3.49 ± 0.77 µM and 4.2 ± 0.74 µM in TRPV1^+/+^ and TRPV1^−/−^ respectively (N = 6, n = 360, 470). There was no significant difference in the sensitivity to ATP between PMUC from TRPV1^+/+^ and TRPV1^−/−^ animals. Interestingly direct application of capsaicin to PMUC failed to elicit a rise in intracellular calcium (fig. 5c) (n = 3, n = 57) despite robust responses to ATP.

### Mediator release

ATP was released from the urothelium both at rest and in response to distension (Fig. 5d). In the present study we found that the stretch evoked ATP release observed in TRPV1^+/+^ mice (4.1 ± 0.9 Vs 14.4 ± 3 nM p ≤ 0.01 n = 6) was absent in the TRPV1^−/−^ mice. In contrast the release of acetylcholine at rest or during bladder distension was unchanged in TRPV1^−/−^ mice (p ≥ 0.05 compared to TRPV1^+/+^ basal and stretch, n = 6) (Fig. 5e).

## Discussion

A number of previous studies have shown that P2X3 sensitive afferent nerves are essential for normal bladder function and that purinergic responses in sensory neurons are largely mediated by P2X2, P2X3 and P2X2/3 receptors^5,6^. Moreover, P2X receptors have been shown to co-localise on afferents expressing TRPV1^30,31^, and co-immunoprecipitation studies have demonstrated a physical association of TRPV1 and P2X3 receptors in the DRG^32^. P2X3 immunoreactivity on bladder projecting neurons is significantly increased in patients with neurogenic detrusor overactivity^33^ and is significantly reduced in those patients who responded positively to intravesical application of the TRPV1 agonist, resiniferatoxin, which causes receptor desensitisation, further suggesting there may also be a functional association between the TRPV1 and P2X3 receptors on bladder afferent neurons innervating the bladder^33^. The results presented in this study provide the first direct evidence that the TRPV1 receptor is able to influence bladder afferent responses to P2X stimulation, a process considered essential to mechanosensation during bladder filling.

αβMe-ATP is a non-selective agonist which activates P2X1 and p2X3 receptor subtypes. Previous studies have provided compelling evidence that P2X1 receptors located on the smooth muscle mediate bladder contraction^34^ and P2X3 receptors located on the afferent nerves play a role in mechanosensation^4,6^. Bladders from wildtype mice responded to the application of αβMe-ATP with a transient bladder contraction and a profound afferent response. In calcium free Krebs, bladder contractions were lost but nerve firing persisted, albeit diminished. This suggests that the receptors responsible for these two components are distinct, and, given the body of evidence in the literature is seems likely that P2X1 receptors on the muscle mediate the contractile response while P2X3 receptors^34^ at the nerve terminal drive the direct nerve response. Membrane bound ionotropic P2X1 and P2X3 receptors, when bound by ATP, mediate non-selective cation influx of Na^+^, and Ca^2+^, and the efflux of K^+^ across the cell membrane under physiological ionic distribution to initiate cell depolarisation^35^. The degree of afferent firing that is maintained in Ca^2+^ free buffer in our experiments is likely a consequence of direct Na^+^ induced membrane depolarisation. Whilst P2X1 receptor mediated influx of both Na^+^ and Ca^2+^ influx in smooth muscle cells creates an excitatory junction potential, it is the subsequent opening of voltage gated calcium channels and calcium induced calcium release from intracellular stores which create the concentrations of intracellular calcium necessary for smooth muscle contraction^36^. Afferent responses to αβMe-ATP were also tested in the presence of the L-type calcium channel blocker nifedipine. Nifedipine significantly attenuated bladder muscle contraction with no effect on afferent firing (unpublished data), further supporting differential dependence of voltage gated calcium channels for muscle contraction and afferent firing in response to P2X receptor activation.

Loss of TRPV1 reduced the bladder afferent response to αβMe-ATP application. It is possible that P2XR expression on bladder afferent endings is altered in the TRPV1^−/−^, thereby affecting the sensitivity to αβMe-ATP. However, the effect of TRPV1 deletion was replicated by the acute treatment with capsazepine, indicating that the attenuated response to αβMe-ATP is unlikely due to a reduction in P2X receptor expression. The similar attenuation observed in the TRPV1^−/−^ and capsazepine treated wildtype mice is also consistent with capsazepine’s pharmacological antagonism of the TRPV1 receptor rather than as a consequence of any non-specific or smooth muscle actions. Interestingly, knockout or pharmacological blockade of the TRPV1 receptor had no effect on detrusor muscle contraction stimulated by αβMe-ATP suggesting that the interaction was at the level of the afferent nerve or urothelium rather than secondary to changes in muscle tone. The effect of capsazepine on afferent firing persists after contractile responses are attenuated in calcium free conditions, again ruling out any effect on bladder contractility. These results are supported by a previous study from our laboratory showing that mechanosensitive responses to ramp distension are significantly attenuated by removal or block of TRPV1 with no concurrent effect on bladder compliance during ramp distension^21^.

TRP channels have been recently shown to modulate the sensitivity of other ion channels^37^ through extensive phosphorylation of intracellular signalling molecules, which in the case of TRPV1 and P2X3/P2X2/3 have been shown to be regulated by the phosphoinositides PiP_2_ and PiP_3_ in peripheral neurons^38,39^. Thus it is possible that removal or blockade of the TRPV1 receptor reduces the sensitivity of P2X3 receptors by alterations to its intracellular signalling cascade.

A previous study into the role of TRPV1 in intestinal mechanosensitivity suggested that loss of TRPV1 function leads to an overall reduction in the pre-tuning of the afferents, thereby reducing excitability^40^. In order to test whether this would explain the phenomenon observed in our study we examined the effect of TRPV1 knockout on the bladder afferent responses to a nicotinic agonist, DMPP, as nicotinic agonists have previously been shown to stimulate capsaicin sensitive bladder afferents^41^. The rationale behind this was to stimulate the bladder by a non-purinergic mechanism in order to assess whether the loss of TRPV1 caused a reduction in nerve sensitivity to both agonists or if the response was specific for αβMeATP. We found no significant difference in the response to DMPP between the wild-type and knock-out mice suggesting that loss of TRPV1 did not result in an overall loss of bladder sensitivity, but that inhibited P2X receptor responses in the TRPV1 knockout is due to a specific interaction between the TRPV1 and P2X3 receptors.

### Dorsal root ganglia

To investigate this relationship in the absence of other potentially confounding factor in the bladder wall, responses to αβMeATP were determined in primary cultures of DRG neurons from lumbosacral (L6/S2) and thoracolumbar (T11/L2) regions from TRPV1^+/+^ and TRPV1^−/−^ mice. DRG neurons from TRPV1^+/+^ responded to αβMe-ATP with an increase in intracellular calcium with similar proportions to those reported by others^42^. We found that the magnitude of the calcium signal was not different between neurons from TRPV1^+/+^ and TRPV1^−/−^ mice. The opening of P2X receptors following αβMe-ATP binding permits the influx of Na^+^, as well as Ca^2+^ ions. Membrane depolarization of sensory neurons is dependent on Na^+^ influx, and results in the opening of voltage-gated calcium channels to act as a second messenger of electric signalling^36^. Sensory neuronal signalling through calcium influx is further amplified by calcium-induced calcium release from intracellular stores which is much larger than the initial calcium influx through P2X receptor ion channels. Thus, it is likely that any difference in P2X-receptor excitability between TRPV1^−/−^ and TRPV1^+/+^ mice is masked by the much larger calcium signals associated with the opening of calcium channels. The proportion of DRG neurons responding to αβMe-ATP was significantly reduced in the TRPV1^−/−^ neurons compared to the wildtypes. In a previous study, Kiyatkin *et al*. found that 30% of DRG neurons exhibit functional responses to both αβMe-ATP and capsaicin confirming that a subpopulation of neurons in the DRG co-express functional TRPV1 and P2X3 receptors^43^. We found the number of responsive DRG neurons in the TRPV1^−/−^ mice was reduced by about 25%, which likely represents the proportion of co-expressing cells in the wild type animals whose absence in the knockout animals would account for the attenuated *ex-vivo* afferent firing in response to αβMe-ATP. Since a similar attenuation was seen following the acute (30 min) treatment with capsazepine it would imply that P2X receptor expression is unchanged in the knockout but sensitivity to ligand is blunted. Together this data suggests that TRPV1 interacts with the P2X3 receptor to modify its sensitivity to agonist stimulation rather than the loss of TRPV1 alters P2X3 receptor expression and further experiments are planned to determine if this is indeed the case. The exact mechanisms involved in this interaction and down-stream signalling pathway still remain to be determined.

### The urothelium

The bladder urothelium plays a key role in modulating the excitability of bladder afferent nerves. This is achieved by the non-neuronal release of inhibitory (such as NO) and excitatory (such as ATP) mediators. In this study, we examined whether the absence of TRPV1 also influenced the ability of the urothelium to respond to purinergic receptor stimulation or alter transmitter release. In concordance with a number of previous studies, we found no evidence that capsaicin caused a calcium response from urothelial cells^14,15,18^ suggesting that TRPV1 may not be functionally coupled to calcium entry or release from intracellular stores in the mouse urothelium, at least under baseline conditions. Paradoxically, however, ATP release from the bladder was attenuated in the TRPV1^−/−^ mouse, an effect which has previously been reported using isolated rat urothelial cells^22^.

As such, the role of TRPV1 in the urothelium remains controversial, and the detection of a functional TRPV1 receptor from a range of species, including human, appears to be dependent on the specific experimental procedures used, and perhaps an over reliance on immunocytochemistry in the absence of confirmatory assays^14–20^.

In the present study TRPV1 mRNA was identified in the urothelium, albeit in low levels, suggesting that TRPV1 might be expressed in urothelial cells despite the failure of capsaicin to evoke a response. This suggests that the levels of TRPV1 mRNA translation may be insufficient to elicit a calcium response, yet high enough to affect ATP release. Immunohistochemical localisation of TRPV1 in the urothelium has been questioned, citing a lack of knockout studies and relevant controls^44^. Genetic deletion of TRPV1 can lead to compensatory changes that attenuate ATP release. Birder *et al*. (2002) showed that capacitance of the urothelium was blunted in the TRPV1^−/−^ mouse suggesting that urothelial trafficking mechanisms might be disrupted following deletion of TRPV1^22^. Importantly, however there was no difference in the sensitivity of PMUCs to ATP as indicated by similar pEC50s suggesting that the interaction between TRPV1 and purinergic receptors observed in the afferent recordings are unlikely to be due to a mechanism involving the urothelium.

In contrast to ATP, acetylcholine release was unaffected by the loss of TRPV1. This was surprising since several previous studies have shown that ATP mediates a positive feedback mechanism on ACh outflow from the urothelium^45,46^. The fact that ATP release was changed by the loss of TRPV1 but that ACh release was not suggests that this feedback mechanism was uncoupled. This may be as a direct result of the differential release mechanism for the two mediators (ie ATP is released vesicularly in a graded manner, whereas ACh is released independent of vesical trafficking in an all-or-nothing manner)^47^ or a developmental compensation aimed at maintaining control over micturition. However, until further studies are conducted to understand how these two transmitters are coupled interpreting this data can only be speculative.

### Concluding remarks

The TRPV1 receptor may have two synergistic roles in mediating bladder mechanosensitivity. A modulatory role in regulating the release of ATP from the urothelium, coupled with a decrease in afferent nerve sensitivity to ATP could combine to result in the deficit in mechanosensitivity observed during bladder distension^21^. Importantly it has been shown that the relative density of suburothelial P2X3 and TRPV1 immunoreactive nerves increases in disease^28,33^ and is significantly reduced following successful treatment with intravesical instillation of resiniferatoxin or capsaicin to cause receptor desensitization. This pathway, therefore, may contribute a larger proportion of the mechanosensory response to distension during pathophysiological conditions. The present results provide a mechanistic basis for the decrease in sensory symptoms seen following resiniferatoxin and capsaicin treatment for lower urinary tract symptoms^48,49^, although the relative contribution of the urothelium and the sub-urothelial afferent nerves remains unknown.

## Methods

### Animals

All experiments were performed in accordance with the University of Sheffield’s Animal Care Committee under a project license issued by the UK Animals (Scientific Procedures) Act 1986. TRPV1 wildytpe (TRPV1^+/+^) and TRPV1 Knockout (TRPV1^−/−^) mice with a genetic background of C57/BL6 were obtained from GlaxoSmithKline (Harlow, UK). Trans-membrane domains 2–4 of the mouse VR1 gene (i.e. DNA encoding amino acids 460–555) were replaced by the neo gene^50^. Mating pairs of TRPV1^−/−^ and TRPV1^+/+^ N1F1 littermates were used to generate separate colonies of TRPV1^+/+^ and TRPV1^−/−^ mice at the University of Sheffield. In the course of the study, genotyping was periodically performed to confirm the absence of the TRPV1 gene in the TRPV1^−/−^ mice. There were no overt differences in feeding behaviour, litter size, growth rate and body weight between TRPV1^−/−^ and TRPV1^+/+^ groups.

16–18 week-old male TRPV1^−/−^ and TRPV1^+/+^ animals were used in this study. Maintenance and killing of the animals followed principles of good laboratory practice in compliance with UK national laws and regulations. The mice were killed humanely by cervical dislocation.

### Afferent nerve recording

Nerve recording was conducted using an *in vitro* model previously described^12^. The whole pelvic region was dissected and placed in a recording chamber (30 mls), continually perfused with gassed (95% O_2_, 5% CO_2_) Krebs-bicarbonate solution (composition in mmol/L: NaCl 118.4, NaHCO_3_ 24.9, CaCl_2_ 1.9, MgSO_4_ 1.2, KCl 4.7, KH_2_PO_4_ 1.2, glucose 11.7) at 35 °C. The urethra and dome were cannulated to enable recording of intravesical pressure and enable evacuation of fluid. Pelvic and hypogastric nerves were dissected into fine multiunit branches, and afferent activity was recorded by a neurolog headstage (NL100, Digitimer Ltd, UK), amplified, filtered (NL215, band pass 300–4000 Hz) and captured by a computer via a power 1401 interface and Spike 2 software (version 7, CED, UK).

### Isolation of mouse dorsal root ganglion (DRG) neurones

DRG’s from pelvic (L6-S1) and hypogastric (L1-L2) regions of the mouse spinal cord were isolated and incubated in HBSS (pH 7.4) containing collagenase (4 mg/ml), and dispase (4.5 mg/ml), at 37 °C for 20 minutes. The collagenase/dispase solution was aspirated and replaced with Hanks balanced salt solution (HBSS) containing papain (2 mg/ml) for 20 minutes at 37 °C. The papain solution was aspirated, replaced with 500 µl DMEM F12 (Gibco) and cells were dissociated via trituration with a Pasteur pipette. Cells were plated onto matrigel coated coverslips (corning) and left for 2 hrs before flooding with DMEM containing 10% FBS and Penstrep (100 IU/ml of penicillin and 100 ug/ml of streptomycin) (Sigma).

### Isolation of mouse urothelial cells

Bladders from TRPV1^−/−^ and TRPV1^+/+^ mice were dissected, pinned urothelial side up and incubated with MEM media containing 2.5 mg/ml dispase (2–3 hours at 21 °C). Cells were collected by gentle scraping and dissociated in 0.025% trypsin EDTA at 37 °C (10 minutes) and re-suspended in keratinocyte serum free media (KSFM) before plating overnight onto collagen (IV) coated coverslips.

### Calcium imaging of cultured urothelial and DRG neurones

Cultured DRG neurones and urothelial cells (20–24 hours) were loaded with 2 μM fura-2-acetoxymethyl ester (fura-2AM) for 30 minutes at 37 °C and washed (Hepes buffer: Hepes 10 mM, NaCl 135 mM, KCl 5 mM, glucose 10 mM, CaCl_2_2 mM and MgCl_2_ 1 mM, pH 7.4) for 1 hr prior to imaging. Cells were stimulated with either ATP, αβMethylene ATP, or capsaicin for 3–5 minutes and changes in intracellular calcium [Ca^2+^]_i,_ were monitored in real-time. Ionomycin (Sigma) (5 µM) was applied as a positive control in DRG experiments. Results are expressed either as relative fluorescence (RF) or normalised to percentage of maximum ionomycin-induced calcium influx to overcome intra-experimental variability. [Ca^2+^]_i_ is represented as the ratio between the fluorescence signal at 340/380 nm. Numbers are quoted as: N = number of mice and n = number of cells.

### Experimental protocols

In nerve recording experiments, the preparation was allowed an equilibration period of 30 min before any experimental protocol commenced. Control bladder distensions were always carried out using isotonic saline (NaCl, 0.9%) at a rate of 100 µl min^−1^ to a maximum pressure of 40 mmHg; at this pressure the infusion pump was stopped and rapid evacuation of the fluid occurred by opening the two-way catheter in the dome. This was repeated several times at intervals of 10 min to assess the viability of the preparation and reproducibility of the intravesical pressure and neuronal responses to distension. Following equilibration, bladders were partially filled (12 mmHg) with the outflow tap closed and left to equilibrate until bladder pressure and baseline nerve activity were stable (approx. 45 mins). αβMethylene-ATP (30 µM) or dimethyl-4-phenylpiperazinium (DMPP) (100 µM) were applied to the bath by bolus dose and washed out with Krebs buffer after 60 seconds with the perfusion system. Krebs buffer was continually perfused for one hour to minimize receptor desensitisation before a second administration of experimental compound. In some experiments, capsazepine (CPZ) (10 µM), or vehicle control (0.01% DMSO) was perfused in the Krebs solution for the period prior to the second stimulus. Bladder contraction was measured as a change in intravesical pressure (mmHg) and latency was calculated as the time (in seconds) from drug application to maximal nerve firing.

### Mediator release

Sequential filling (ramp distension) of a catheterised bladder (as described above) was performed (0.9% NaCl, to 40 mmHg). Intraluminal fluids were collected and the amount of ATP and Acetylcholine (ACh) in the samples was determined using the luciferin-luciferase ATP bioluminescent assay and the Amplex red ACh/ACh esterase assay kit (Molecular Probes).

### qPCR

mRNA from cultured urothelial cells and DRG neurones (20–24 hrs) was isolated (RNAeasy minikit, Qaigen) and cDNA was synthesised by reverse transcription using superscript III (Invitrogen) from mRNA following the manufacturers protocol. cDNA was amplified by PCR for 35 cycles with the following primers and Sybr green fluorescence (Biorad). Primer sequence for the TRPV1 receptor was forward 5′ CAAGGCTCTATGATCGCAGG 3′ and reverse 5′GAGCAATGGTGTCGTTCTGC 3′. All samples were assayed in triplicate in the same plate. The relative amount of target mRNA was calculated by the 2^−ΔΔCt^ method using β-actin as a housekeeping gene.

### Data analysis and Statistics

Whole nerve multi-fibre afferent nerve activity was quantified using a Spike2 software which counted number of action potentials crossing a pre-set threshold (Cambridge Electronic Design, Cambridge, UK).

For afferent nerve recording experiments, baseline activity was subtracted from stimulus induced response. Data are presented as means ± S.E.M. Statistical analysis was carried out using either a 1 or 2-way ANOVA and appropriate post-hoc test or a Student’s T-test, where appropriate. Tukey post-hoc test was used for 2-way ANOVA when n values for groups was the same, and Bonferroni for all other ANOVA, with significance confirmed at P < 0.05.

## References

[1] Groat, W. & Yoshimura, N. in *Sensory Nerves* Vol. 194 *Handbook of Experimental Pharmacology* (eds Brendan J. Canning & Domenico Spina) Ch. 4, 91–138 (Springer Berlin Heidelberg, 2009).

[2] Pandita RK, Andersson K-E (2002). Intravesical adenosine triphosphate stimulates the micturition reflex in awake, freely moving rats. The Journal of Urology.

[3] Namasivayam S, Eardley I, Morrison JF (1999). Purinergic sensory neurotransmission in the urinary bladder: an *in vitro* study in the rat. BJU International.

[4] Rong W, Spyer KM, Burnstock G (2002). Activation and sensitisation of low and high threshold afferent fibres mediated by P2X receptors in the mouse urinary bladder. The Journal of Physiology.

[5] Cockayne DA (2005). P2X2 knockout mice and P2X2/P2X3 double knockout mice reveal a role for the P2X2 receptor subunit in mediating multiple sensory effects of ATP. The Journal of Physiology.

[6] Vlaskovska M (2001). P2X3 knock-out mice reveal a major sensory role for urothelially released ATP. The Journal of Neuroscience.

[7] Svennersten K, Hallén-Grufman K, de Verdier PJ, Wiklund NP, Poljakovic M (2015). Localization of P2X receptor subtypes 2, 3 and 7 in human urinary bladder. BMC Urology.

[8] Bayliss M, Wu C, Newgreen D, Mundy AR, Fry CH (1999). A quantitative study of atropine-resistant contractile responses in human detrusor smooth muscle, from stable, unstable and obstructed bladders. J Urol.

[9] Liu F, Takahashi N, Yamaguchi O (2009). Expression of P2X3 purinoceptors in suburothelial myofibroblasts of the normal human urinary bladder. International Journal of Urology.

[10] Apostolidis A (2005). Decreased sensory receptors P2X3 and TRPV1 in suburothelial nerve fibers following intradetrusor injections of botulinum toxin for human detrusor overactivity. The Journal of Urology.

[11] Tempest, H. V. *et al*. P2X and P2X receptor expression in human bladder urothelium and changes in interstitial cystitis. *BJU Int* **93**, 10.1111/j.1464-410X.2004.04858.x (2004). 10.1111/j.1464-410X.2004.04858.x 15180635

[12] Laing RJ, Dhaka A (2016). ThermoTRPs and Pain. The Neuroscientist: a review journal bringing neurobiology, neurology and psychiatry.

[13] Caterina MJ (1997). The capsaicin receptor: a heat-activated ion channel in the pain pathway. Nature.

[14] Shabir S (2013). Functional expression of purinergic P2 receptors and transient receptor potential channels by the human urothelium. American Journal of Physiology - Renal Physiology.

[15] Yu W, Hill WG (2011). Defining protein expression in the urothelium: a problem of more than transitional interest. American Journal of Physiology - Renal Physiology.

[16] Everaerts W (2010). Functional characterization of transient receptor potential channels in mouse urothelial cells. American Journal of Physiology - Renal Physiology.

[17] Birder LA (2010). Urothelial signaling. Autonomic Neuroscience.

[18] Yamada T (2009). *Differential localizations of the transient* receptor potential channels TRPV4 and TRPV1 in the mouse urinary bladder. Journal of Histochemistry & Cytochemistry.

[19] Xu X (2009). Functional TRPV4 channels and an absence of capsaicin-evoked currents in freshly-isolated, guinea-pig urothelial cells. Channels.

[20] Charrua A (2009). Functional transient receptor potential vanilloid 1 is expressed in human urothelial cells. The Journal of Urology.

[21] Daly D, Rong W, Chess-Williams R, Chapple C, Grundy D (2007). Bladder afferent sensitivity in wild-type and TRPV1 knockout mice. The Journal of Physiology.

[22] Birder LA (2002). Altered urinary bladder function in mice lacking the vanilloid receptor TRPV1. Nat Neuroscience.

[23] Avelino A, Cruz C, Nagy I, Cruz F (2002). Vanilloid receptor 1 expression in the rat urinary tract. Neuroscience.

[24] Cefalu JS (2009). Selective pharmacological blockade of the TRPV1 receptor suppresses sensory reflexes of the rodent bladder. The Journal of Urology.

[25] Charrua A (2009). GRC-6211, a new oral specific TRPV1 antagonist, decreases bladder overactivity and noxious bladder input in cystitis animal models. The Journal of Urology.

[26] Charrua A, Cruz CD, Cruz F, Avelino A (2007). Transient receptor potential vanilloid subfamily 1 is essential for the generation of noxious bladder input and bladder overactivity in cystitis. The Journal of Urology.

[27] Dinis P, Charrua A, Avelino A, Cruz F (2004). Intravesical resiniferatoxin decreases spinal c-fos expression and increases bladder volume to reflex micturition in rats with chronic inflamed urinary bladders. BJU International.

[28] Brady CM (2004). Parallel changes in bladder suburothelial vanilloid receptor TRPV1 and pan-neuronal marker PGP9.5 immunoreactivity in patients with neurogenic detrusor overactivity after intravesical resiniferatoxin treatment. BJU International.

[29] Sadananda P, Shang F, Liu L, Mansfield KJ, Burcher E (2009). Release of ATP from rat urinary bladder mucosa: role of acid, vanilloids and stretch. British Journal of Pharmacology.

[30] Dang K, Bielefeldt K, Gebhart GF (2005). Differential responses of bladder lumbosacral and thoracolumbar dorsal root ganglion neurons to purinergic agonists, protons, and capsaicin. The Journal of Neuroscience.

[31] Piper AS, Docherty RJ (2000). One-way cross-desensitization between P2X purinoceptors and vanilloid receptors in adult rat dorsal root ganglion neurones. The Journal of Physiology.

[32] Stanchev D (2009). Cross-inhibition between native and recombinant TRPV1 and P2X3 receptors. Pain.

[33] Brady CM (2004). P2X3-immunoreactive nerve fibres in neurogenic detrusor overactivity and the effect of intravesical resiniferatoxin. European Urology.

[34] Vial C, Evans RJ (2000). P2X receptor expression in mouse urinary bladder and the requirement of P2X(1) receptors for functional P2X receptor responses in the mouse urinary bladder smooth muscle. British Journal of Pharmacology.

[35] North, R. A. P2X receptors. *Philosophical transactions of the Royal Society of London. Series B, Biological sciences* **371**, 10.1098/rstb.2015.0427 (2016). 10.1098/rstb.2015.0427 PMC4938027 27377721

[36] Hill-Eubanks DC, Werner ME, Heppner TJ, Nelson MT (2011). Calcium Signaling in Smooth Muscle. Cold Spring Harbor Perspectives in Biology.

[37] Harrington AM (2011). A novel role for TRPM8 in visceral afferent function. Pain.

[38] Mo G (2009). Subtype-specific regulation of P2X3 and P2X2/3 receptors by phosphoinositides in peripheral nociceptors. Molecular pain.

[39] Rohacs T, Nilius B (2007). Regulation of transient receptor potential (TRP) channels by phosphoinositides. Pflugers Archiv: European journal of physiology.

[40] Rong W (2004). Jejunal afferent nerve sensitivity in wild-type and TRPV1 knockout mice. The Journal of Physiology.

[41] Masuda, H. *et al*. Roles of Peripheral and Central Nicotinic Receptors in the Micturition Reflex in Rats. *The Journal of Urology* **176**, 374–379, 10.1016/S0022-5347(06)00581-7. 10.1016/S0022-5347(06)00581-7 16753446

[42] Cockayne DA (2000). Urinary bladder hyporeflexia and reduced pain-related behaviour in P2X3-deficient mice. Nature.

[43] Kiyatkin ME, Feng B, Schwartz ES, Gebhart GF (2013). Combined genetic and pharmacological inhibition of TRPV1 and P2X3 attenuates colorectal hypersensitivity and afferent sensitization. American journal of physiology. Gastrointestinal and liver physiology.

[44] Everaerts W (2009). Where is TRPV1 expressed in the bladder, do we see the real channel?. Naunyn-Schmied Arch Pharmacol.

[45] Sui G (2014). Purinergic and muscarinic modulation of ATP release from the urothelium and its paracrine actions. American Journal of Physiology - Renal Physiology.

[46] Silva-Ramos M (2013). Urinary ATP may be a dynamic biomarker of detrusor overactivity in women with overactive bladder syndrome. PLoS ONE.

[47] McLatchie LM, Young JS, Fry CH (2014). Regulation of ACh release from guinea pig bladder urothelial cells: potential role in bladder filling sensations. Br J Pharmacol.

[48] Apostolidis A, Gonzales GE, Fowler CJ (2006). Effect of intravesical resiniferatoxin (RTX) on lower urinary tract symptoms, urodynamic parameters, and quality of life of patients with urodynamic increased bladder sensation. European Urology.

[49] Silva C, Silva J, Ribeiro M-J, Avelino A, Cruz F (2005). Urodynamic effect of intravesical resiniferatoxin in patients with neurogenic detrusor overactivity of spinal origin: results of a double-blind randomized placebo-controlled trial. European Urology.

[50] Davis JB (2000). Vanilloid receptor-1 is essential for inflammatory thermal hyperalgesia. Nature.

